# Characterization of transcriptome profile and clinical features of a novel immunotherapy target CD204 in diffuse glioma

**DOI:** 10.1002/cam4.2312

**Published:** 2019-05-29

**Authors:** Yongliang Yuan, Qitai Zhao, Songfeng Zhao, Penghua Zhang, Haibiao Zhao, Zeyun Li, Yue Du, Xin Tian, Jingli Lu

**Affiliations:** ^1^ Department of Pharmacy The First Affiliated Hospital of Zhengzhou University Zhengzhou China; ^2^ Henan Key Laboratory of Precision Clinical Pharmacy Zhengzhou University Zhengzhou China; ^3^ Biotherapy Center The First Affiliated Hospital of Zhengzhou University Zhengzhou China; ^4^ Department of Oncology The First Affiliated Hospital of Zhengzhou University Zhengzhou China; ^5^ Imaging Department The Third Affiliated Hospital of Zhengzhou University Zhengzhou China; ^6^ Department of Neurosurgery The First Affiliated Hospital of Zhengzhou University Zhengzhou China

**Keywords:** biomarker, CD204, glioma, immune checkpoints, prognosis

## Abstract

CD204 is a specific marker of tumor‐associated macrophages (TAMs) in glioma. However, the expression levels of CD204 and its involvement in glioma are not fully understood. In this large‐scale study, we assessed the expression and function of CD204 in whole‐grade glioma molecularly and clinically. In total, 1323 glioma samples, including 301 microarray data and 325 RNA‐seq data from the Chinese Glioma Genome Atlas (CGGA) dataset and 697 RNA‐seq data from The Cancer Genome Atlas (TCGA) dataset, were utilized. The statistical analysis and graphical work were mainly performed using the R software. Univariate and multivariate Cox analysis demonstrated that CD204 was an independent prognosticator in glioma patients. CD204 expression was positively correlated with the grade of malignancy. CD204 was consistently upregulated in wild‐type isocitrate dehydrogenase glioma and highly expressed in mesenchymal glioblastoma. Gene ontology of CD204‐related genes showed that CD204 was most enriched in inflammatory response and immune response. It was associated with the stromal and immune populations, especially the monocytic lineage, fibroblasts, and T cells. Circos plots revealed that CD204 was closely associated with many immune checkpoint regulators, especially TIM‐3. CD204 expression is consistent with the malignant phenotype of glioma and independently predicts poor outcomes in glioma patients. Additionally, CD204^+^ TAMs, collaborating with other checkpoint members, may contribute to the dysfunction of T cells. These findings suggest that CD204 may be a promising target for glioma immunotherapy.

## 1 INTRODUCTION

Glioma is the most common primary cancer of the central nervous system, accounting for approximately 50% of all primary brain tumors, and it has been classified into grades I to IV by the World Health Organization (WHO).^1^, ^2^ Despite the improvements in treatment, glioblastoma multiforme (GBM, WHO grade IV), the most malignant and aggressive form of glioma, remains a leading cause of death among human cancers, having a median survival time of less than 15 months.^3^ Considering the poor outcome after classical treatment, there is a dire need for new therapeutic approaches.

Increasing amounts of evidence indicate that the tumor microenvironment plays a vital role in supporting the progression of glioma. It consists of stromal cells and immune cells, including fibroblasts, endothelial cells, tumor‐infiltrating lymphocytes (TILs), natural killer (NK) cells, dendritic cells (DCs), tumor‐associated macrophages (TAMs), myeloid‐derived suppressor cells (MDSCs), and regulatory T cells (Tregs).^4^ Of these, TAMs are thought to play a major role and account for nearly 30% of the tumor mass.^5^ They consist of three subpopulations depending on the source of the cells, namely brain‐resident microglia, bone marrow‐derived macrophages (BMDMs), and extra‐parenchymal macrophages.^6^ Unlike classically activated M1 macrophages, which are characterized by their promotion of an antitumor response, TAMs have an immunosuppressive role, thus being defined as the alternatively activated M2 phenotype.^7^, ^8^, ^9^ TAMs promote glioma progression through secretion of TGFβ1, IL‐6, VEGF, PDGF, periostin, MMP2, and MMP9, maintaining the phenotype of glioma stem‐like cells, promoting angiogenesis, and regulating degradation of the extracellular matrix (ECM), thus leading to glioma growth, invasion, and the formation of distant pre‐metastatic niches.^10^, ^11^ Moreover, the immunosuppressive microenvironment induced by TAMs suppresses the systemic immune response by blocking T‐cell proliferation, increasing T‐cell apoptosis, inhibiting cytotoxic T lymphocyte (CTL) response, and promoting other immunosuppressive cells such as MDSCs and Tregs.^12^, ^13^ Given the paramount role of TAMs in promoting glioma progression, targeting TAMs may prove a successful approach for suppressing the progression of the tumor.

CD204, also known as macrophage scavenger receptor 1 (MSR1),^14^ is considered a specific marker of TAMs in glioma. CD204 is involved in multiple metabolic processes of macrophages, including adhesion, phagocytosis, production of reactive oxygen species, and host defense.^15^ Additionally, CD204^+^ TAMs attenuate the activation of T cells by downregulating toll‐like receptor 4 signaling. In glioma microenvironment, CD204 is alternatively expressed in TAMs and is correlated with the histological grade and outcome of glioma, suggesting it may serve as an immunotherapeutic target.^16^ However, the details of CD204 expression and its role in glioma are still unclear.

In this study, we aimed to assess the expression and immune features of CD204 in glioma based on data from the Chinese Glioma Genome Atlas (CGGA) and The Cancer Genome Atlas (TCGA) datasets. In particular, we assessed the prognostic value of CD204 in glioma, its role and expression in various types of glioma, and its correlation with various functional aspects of the immune system. To the best of our knowledge, this is the first comprehensive study exploring CD204 in glioma both molecularly and clinically via a large‐scale analysis.

## 2 METHODS AND MATERIALS

### 2.1 Data collection

Microarray data and RNA‐seq data with corresponding clinical information of diffuse glioma patient samples were obtained from the CGGA (http://www.cgga.org.cn/) and TCGA (http://cancergenome.nih.gov) databases. In total, 301 microarray and 325 RNA‐seq data from the CGGA dataset as well as 697 RNA‐seq data from the TCGA dataset of diffuse glioma, which ranged from grades II to IV, were assessed in our study.

### 2.2 Human tumor samples

All human glioma tissue samples were obtained from the First Affiliated Hospital of Zhengzhou University. The surgical samples were immediately frozen in liquid nitrogen and stored at − 80°C until further use. WT or IDH1 mutation glioma tissues were identified.

### 2.3 Isolation of PBMCs and co‐culture model

Peripheral blood mononuclear cells (PBMCs) were isolated by Ficoll‐Hypaque density gradient centrifugation, sequentially using the anti‐CD8 MACS magnetic sorting system (Miltenyi Biotec,Germany) within 2 hours of sample collection. Then, the cells were co‐cultured with CD14^+^CD204^+^ cells isolated from tumor tissue cells at 1:2 ratios. After 48 h, the CD8^+^ cells were isolated to perform FACS analysis.

### 2.4 Quantitative real‐time PCR

Total RNA was extracted from cultured cells or fresh tissue samples using Trizol (Invitrogen Corporation, Carlsbad, CA, USA，#A33250). NanoDrop 2000 (Thermo Scientific, Waltham, MA, USA) was used to detect the concentration and purity of total RNA. Then, the mRNA was reversely transcribed into complementary DNA (cDNA) with Prime Script RT reagent kit concluding genomic DNA eraser (TaKaRa, Tokyo, Japan, #RR037A). Primers used for this experiment were listed in the Table S1, all the primers were purchased from Sangon Biotech (Shanghai, China). Each experiment was performed in triplicate, and glyceraldehyde‐3‐phosphate dehydrogenase (*GAPDH*) was used for normalization of data.

### 2.5 Flow cytometry analysis

The cells were collected by centrifugation and washed twice with PBS. Then cells were stained with anti‐human CD14 antibody (BioLegend, San Diego, CA, #367111), CD204 (BioLegend, #371902), CD8 (BioLegend, #371902), IFN‐γ (BioLegend, #506537) at 4℃ for 15 minutes. The samples were analyzed by FACSCanto II flow cytometer (Biolegend, # 302507).

### 2.6 Statistical analysis

The statistical software R, version 3.5.1 (http://www.r-project. org), SPSS software (version 16.0; SPSS Inc, Chicago, IL), and GraphPad Prism 7 software (version 5.0; San Diego, CA) were used for the statistical analysis and generation of figures. The optimal cut‐off point of CD204 expression value was divided into high‐expression group and low‐expression group using the "cutp" function from the R package "survMisc," and the R package “survival” was used to perform Kaplan‐Meier analysis. The univariate and multivariate analyses were performed using coxph function provided in “survival” package, and the receiver operating characteristic (ROC) curves were performed with ROC function. We applied the heatmap to analyze the gene ontology (GO) of the most correlated genes. The pathway activity estimates were obtained using the GSVA package. The ESTIMATE analysis was performed to evaluate the relationship between CD204 and immune populations, while the MCP analysis was utilized to evaluate the relationship between CD204 and tumor microenvironment. The circos plot and the corrgram plot were achieved using Pearson's correlation analysis. Other R packages, such as “ggplot2,” “dplyr,” and “ggsci”, were also applied for visualizing the results of data analysis. For all statistical methods, the two tailed unpaired *t* tests were used to compare the difference of relative gene expression, the results were presented as mean ± SD; each experiment has three biological repetitions. *P *< 0.05 was considered as the significant difference. All statistical tests were two‐sided.

## 3 RESULTS

### 3.1 CD204 is a specific marker of TAMs and is correlated with unfavorable outcome of glioma

Several markers have been utilized to identify TAMs, namely CD204, CD163, CD11b, CD14, and CD68. However, the correlation of these markers in glioma is not fully understood. Therefore, Pearson correlation analysis of these markers in whole‐grade glioma was first performed by analyzing RNA‐seq data in the CGGA dataset and further validated using the TCGA dataset. A high correlation of these markers was observed in both datasets (Figure S1A and B). Moreover, these markers showed higher correlation in GBM (Figure S1C and D). Next, to identify the most representative marker of TAMs in glioma, univariate and multivariate analyses were performed to evaluate the effects of these markers (data not shown), revealing CD204 as the only independent prognosticator in glioma patients in both datasets (Table 1).

**Table 1:** Univariate and multivariate Cox analysis of clinical prognostic parameters in TCGA and CGGA datasets

Characteristic	TCGA (n = 629)			CGGA (Array, n = 297)			CGGA (Seq, n = 310)		
	HR	95% CI	*P*	HR	95% CI	*P*	HR	95% CI	*P*
Univariate									
Age	1.077	1.065‐1.089	<0.0001	1.041	1.027‐1.055	<0.0001	1.038	1.023‐1.054	<0.0001
Gender	0.852	0.645‐1.125	0.259	0.836	0.610‐1.415	0.264	0.847	0.600‐1.195	0.345
Grade	4.985	3.956‐6.286	<0.0001	2.932	2.398‐3.585	<0.0001	3.447	2.716‐4.452	<0.0001
IDH‐mut	10.155	7.373‐13.987	<0.0001	3.188	2.257‐4.504	<0.0001	4.101	2.858‐5.884	<0.0001
CD204	1.716	1.576‐1.868	<0.0001	1.726	1.502‐1.984	<0.0001	2.010	1.759‐2.297	<0.0001
Multivariate									
Age	1.050	1.036‐1.064	<0.0001	1.016	1.002‐1.030	0.022	1.003	0.987‐1.018	0.740
Gender	0.868	0.649‐1.162	0.342	0.908	0.662‐1.246	0.550	1.008	0.705‐1.443	0.964
Grade	1.404	1.015‐1.942	0.040	2.309	1.838‐2.901	<0.0001	2.456	1.838‐3.281	<0.0001
IDH‐mut	2.807	1.724‐4.572	<0.0001	1.453	0.977‐2.162	0.065	1.282	0.798‐2.059	0.305
CD204	1.268	1.137‐1.414	<0.0001	1.233	1.058‐1.435	0.007	1.466	1.238‐1.736	<0.0001

**Figure 1:**
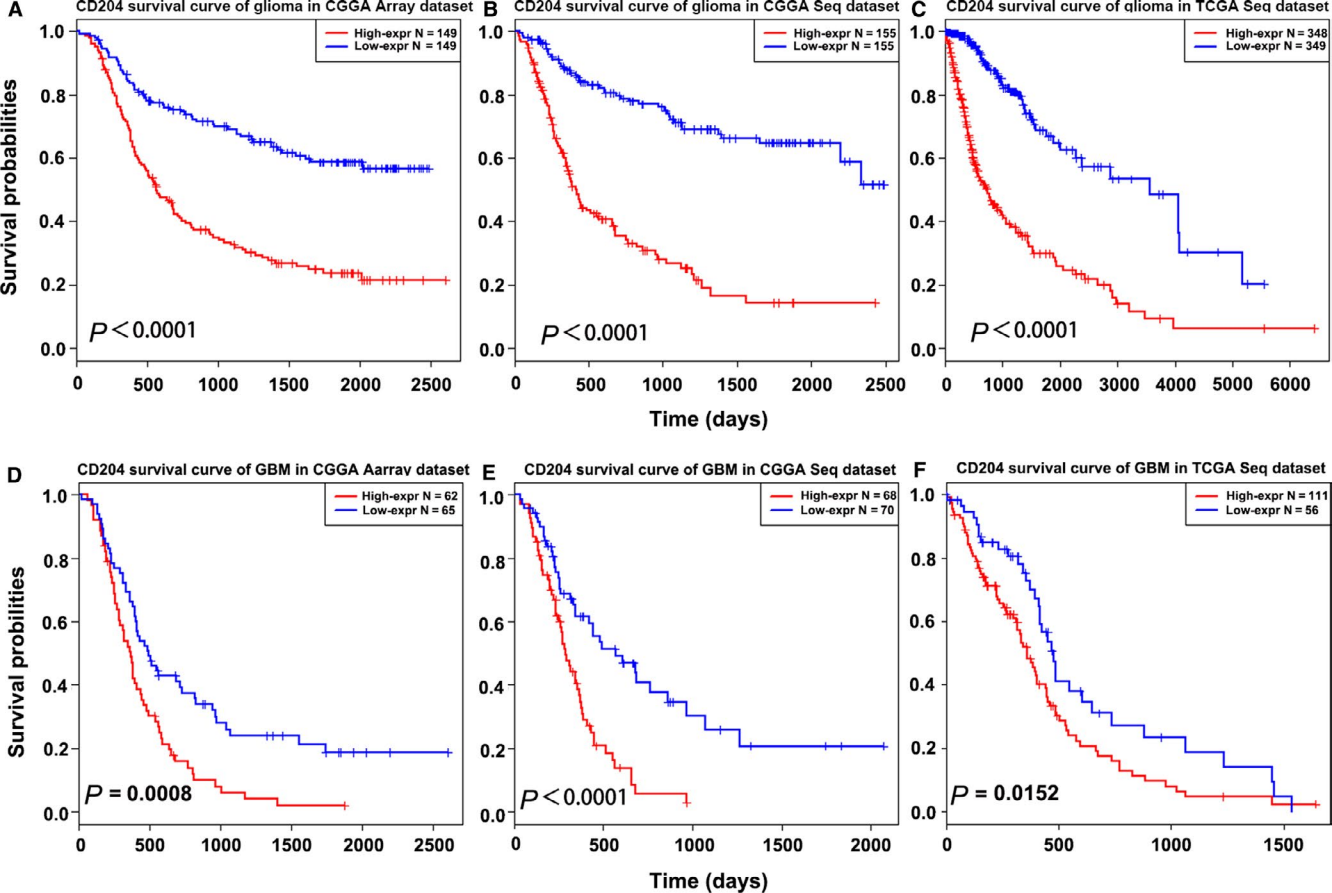
CD204 is correlated with unfavorable overall survival in glioma. (A, B, C) High expression of CD204 predicted worse outcome in whole‐grade glioma patients in the CGGA microarray and RNA‐seq dataset and the TCGA RNA‐seq dataset. The samples were divided into a high‐ and low‐expression group based on the best cut‐off point of CD204 mRNA expression value. (D, E, F) High expression of CD204 predicted worse outcome in GBM in the CGGA microarray and RNA‐seq dataset and the TCGA RNA‐seq dataset

Moreover, the expression level of CD204 in GBM was higher than that in other types of glioma (Figure S2). Kaplan‐Meier analysis revealed that high expression of CD204 predicts a short overall survival (OS) both in the CGGA (Figure 1A and 1) and the TCGA dataset (Figure 1C) in whole‐grade glioma and low‐grade glioma (Figure S3A B and C). Given the high expression of CD204 in GBM, survival analysis in GBM was additionally performed: as expected, the same significance of CD204 was observed (Figure 1D, 1, and 1). We further used Cox's regression models to determine whether the prognostic value of CD204 was independent of other important clinical factors (molecular subtypes, IDH‐mut, grade, age, gender, radiation therapy, MGMT promoter status) in TCGA cohort (n = 411). As shown in the Table S2, multivariate regression analysis showed a significant correlation of CD204 with prognostic factor after adjusting for other clinical factors.

### 3.2 CD204 expression increases with age and glioma grade and is overexpressed in wild‐type IDH glioma

**Figure 2:**
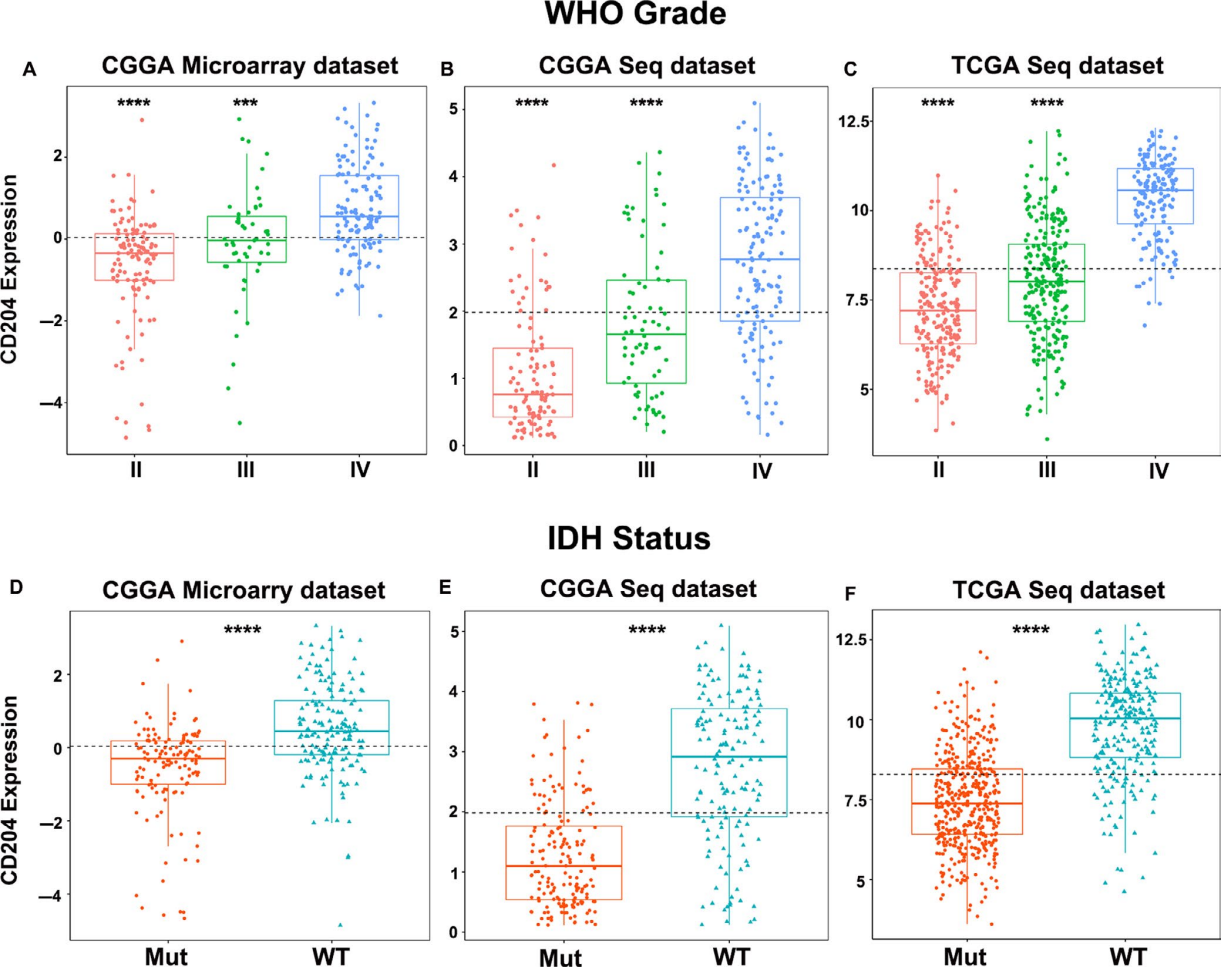
CD204 expression increases with glioma grade and patient age and is overexpressed in wild‐type IDH glioma. (A, B, C) CD204 expression was correlated with glioma grade in the CGGA microarray and RNA‐seq data and the TCGA RNA‐seq data. CD204 expression level was divided based on tumor grade (II–IV). (D, E, F) CD204 was highly expressed in wild‐type IDH glioma in the CGGA microarray and RNA‐seq data and the TCGA RNA‐seq data. The patients were divided into a wild‐type and mutant IDH group, and the difference between CD204 expression in two groups was analyzed using R. *** represents *P *< 0.001, **** represents *P* < 0.0001

Apart from CD204, patient age, tumor grade, and isocitrate dehydrogenase (IDH) status were also found to play important roles in predicting the outcomes of glioma patients. To investigate the correlation of CD204 expression in glioma with these parameters, 626 samples were selected from the microarray and RNA‐seq data of the CGGA dataset. CD204 expression was found to increase with age (Figure S4A, B, and C) and glioma grade in both the CGGA (Figure 2A and 2) and the TCGA dataset (Figure 2C). These results indicate that CD204^+^ TAMs may enhance the immunosuppressive state along with age and tumor progression. As IDH mutations widely occur in glioma and predict a better outcome,^17^ we stratified the data based on IDH status and analyzed CD204 expression in two types of glioma. The results revealed that CD204 was significantly overexpressed in wild‐type IDH glioma (Figure 2D, 2, and 2) in both datasets. There results were in line with clinical sample experiments that CD204 was high expressed in wild‐type IDH glioma samples (Figure S5), indicating that TAMs are more prevalent in wild‐type IDH glioma. Furthermore, ROC curve analysis was performed to further investigate the diagnostic value of CD204 in wild‐type IDH glioma. The area under the curve (AUC) was 88.5%, 82.4%, and 85.6% in the CGGA microarray and RNA‐seq and the TCGA RNA‐seq data, respectively (Figure S6A, B, and C).

### 3.3 CD204 is a potential marker for the mesenchymal subtype of glioblastoma

**Figure 3:**
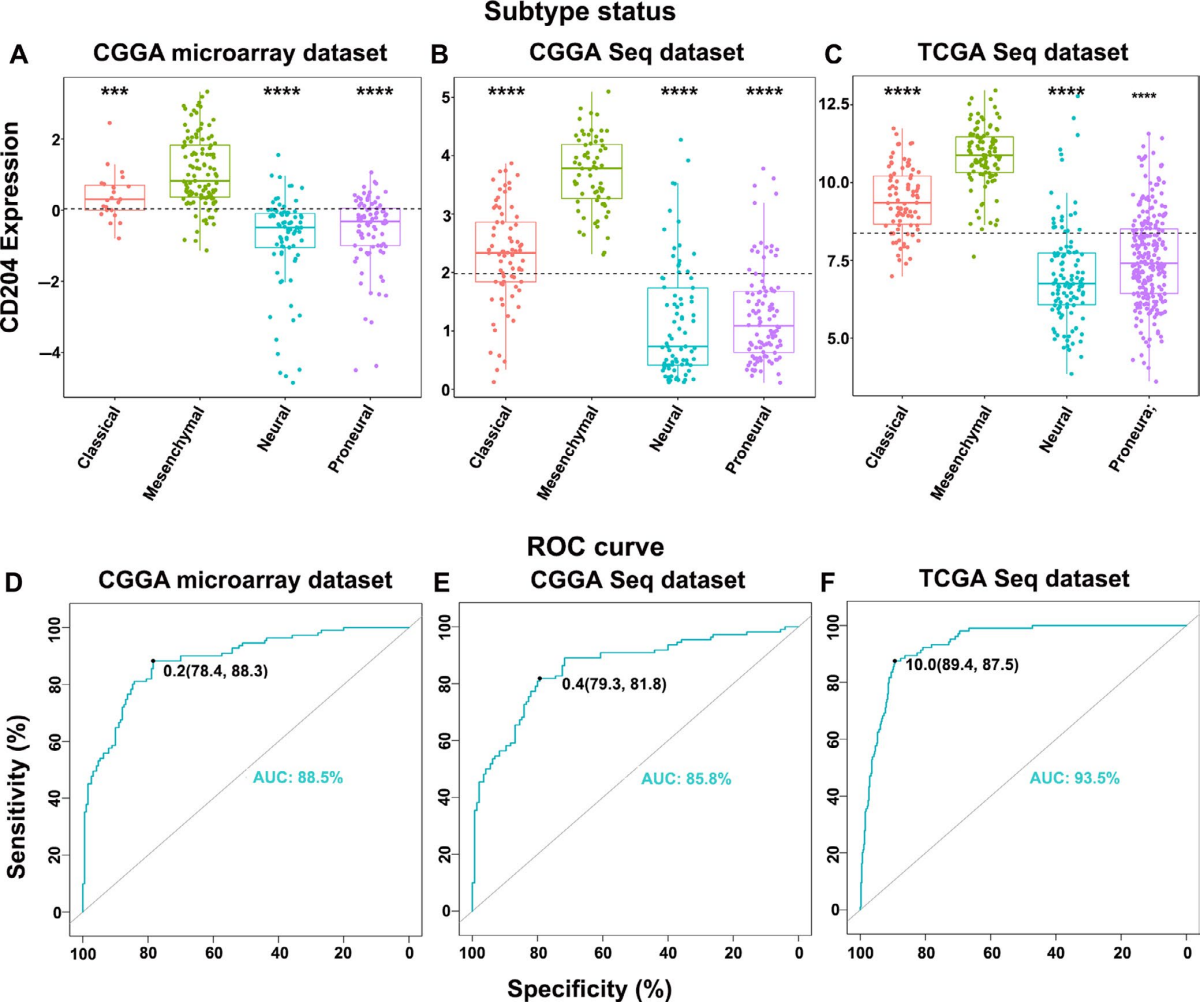
CD204 is a potential marker for mesenchymal glioblastoma. (A, B, C) CD204 expression was higher in mesenchymal glioblastoma than in the other molecular subtypes of glioma. (D, E, F) ROC curve analysis of CD204 in mesenchymal glioblastoma. *** represents *P *< 0.001, **** represents *P *< 0.0001

To gain a better understanding of CD204 expression in glioma, CD204 expression was analyzed in the four subtypes of glioblastoma defined by the TCGA network.^18^ Based on CGGA microarray and RNA‐seq data, CD204 was significantly upregulated in mesenchymal glioblastoma compared to the other molecular subtypes (Figure 3A and 3). The same results were observed by analyzing the TCGA dataset (Figure 3C). To further clarify whether CD204 is specifically overexpressed in mesenchymal glioblastoma, ROC curve analysis of CD204 expression was performed, and the AUC was 88.5%, 85.8%, and 93.5% in the CGGA microarray and RNA‐seq and the TCGA RNA‐seq data, respectively (Figure 3D, 3 and 3).

### 3.4 Biological functions of CD204

**Figure 4:**
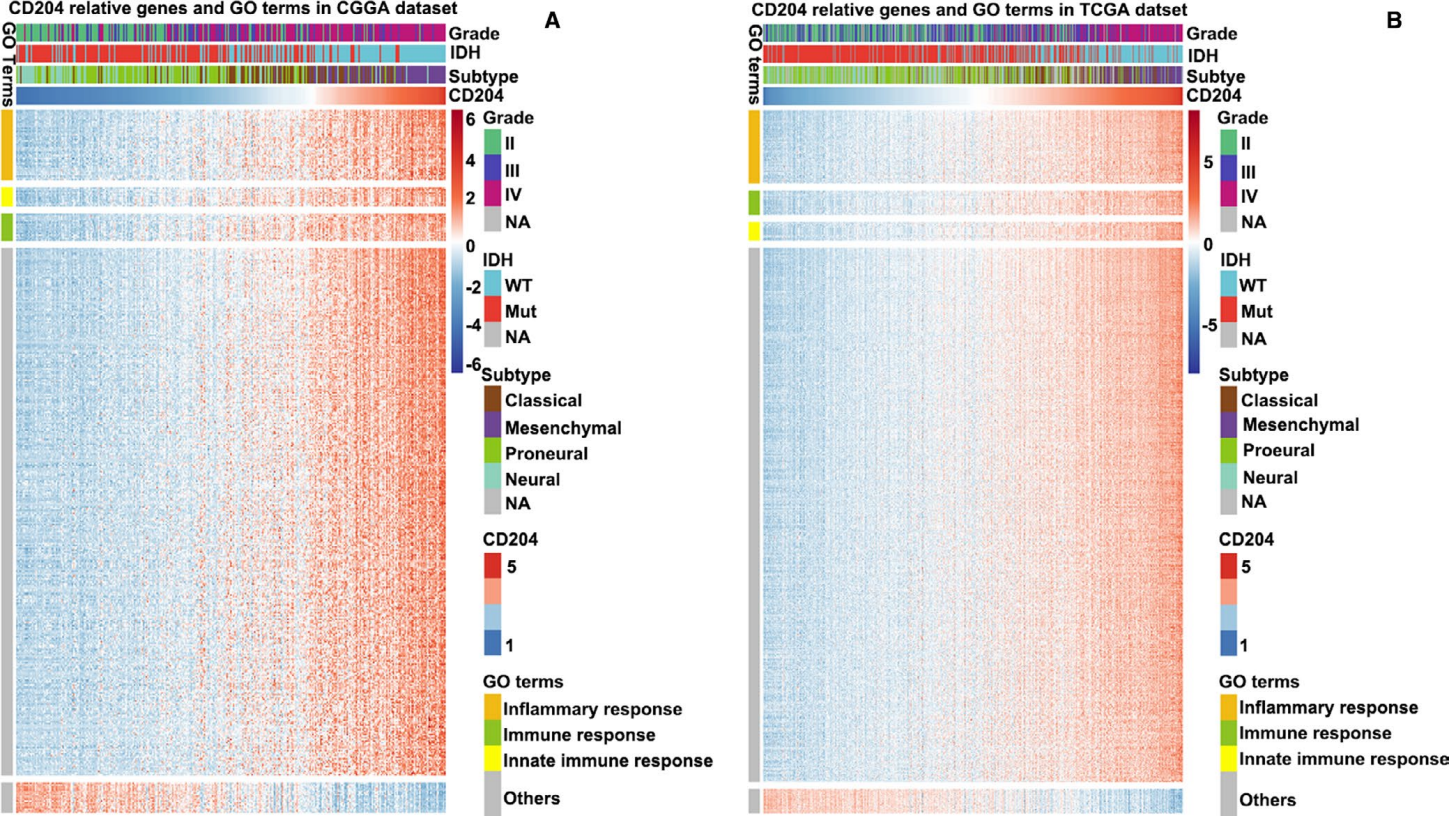
Biological function of CD204. (A, B) CD204‐related biological processes by GO analysis in the CGGA and TCGA datasets. The top 600 genes correlated with CD204 by Person correlation analysis were selected, and GO enrichment analysis was performed on them

Since CD204 expression in glioma was strongly associated with malignancy, we speculated that CD204 may have important biologic functions in glioma. Thus, genes strongly correlated with CD204 expression, that is, the top 600 genes ranked by Pearson's correlation coefficient in the CGGA and TCGA datasets, were selected. Subsequently, GO enrichment analysis of these genes was performed through online tools (DAVID, https://david.ncifcrf.gov/). CD204‐related genes were mainly enriched in inflammatory response, immune response, and the interferon‐gamma‐mediated signaling pathway in both datasets (Figure 4A and 4). Notably, the CD204‐related gene enrichment function was strongly associated with antigen processing and presentation, MHC‐II proteins, and receptor activity (Figure S7A and B).

### 3.5 CD204 in inflammatory response

**Figure 5:**
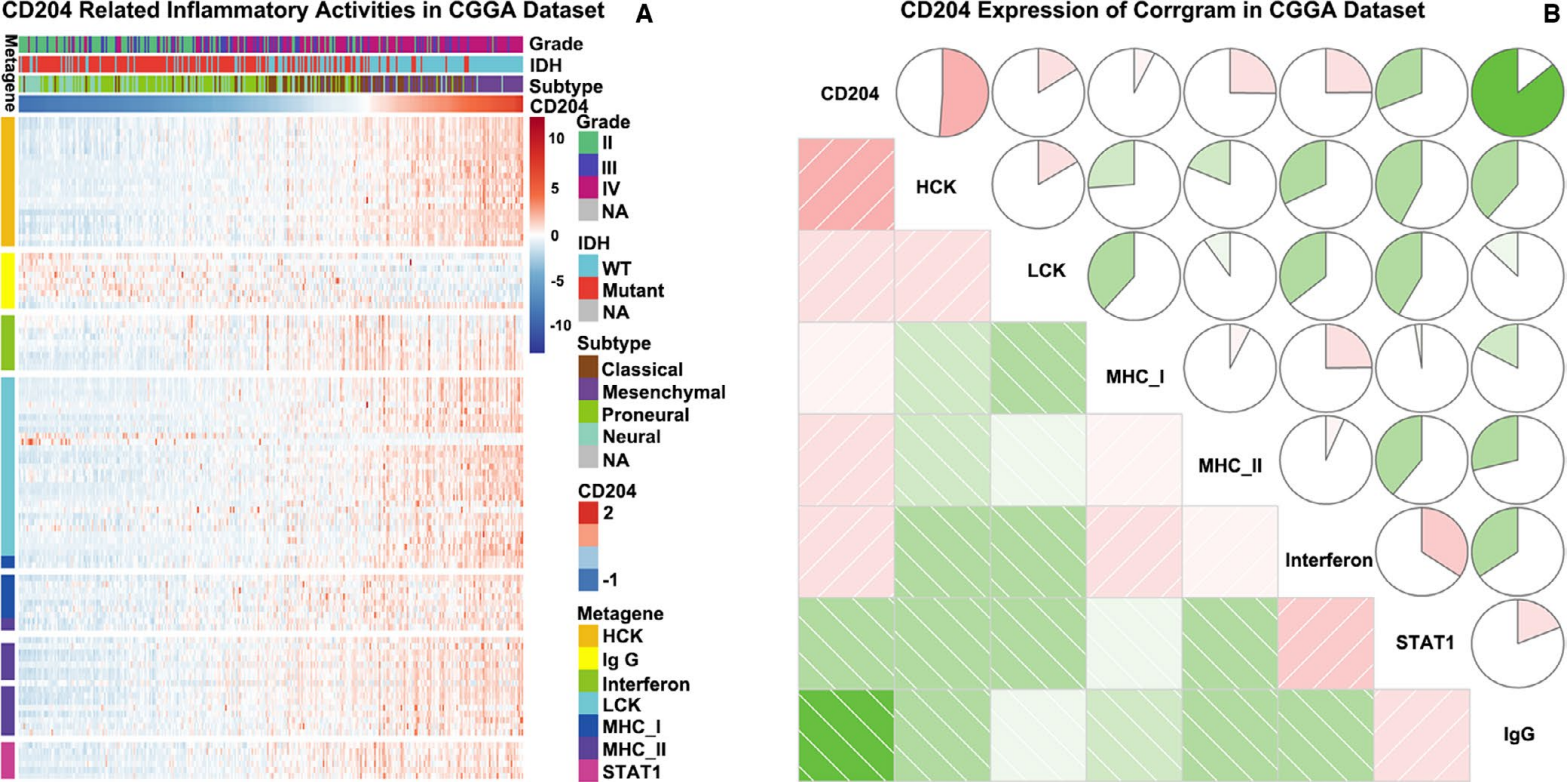
CD204 in inflammatory response. (A) The heatmap of CD204‐related inflammatory metagenes in the CGGA dataset. CD204 was positively correlated with HCK, LCK, and interferon and was negatively correlated with IgG. (B) Correlogram of CD204 and inflammatory metagenes in the CGGA dataset

To further explore how CD204 mediates the inflammatory response, seven clusters of 135 inflammatory response‐related genes were identified in the CGGA dataset. CD204 was positively correlated with the protein tyrosine kinases HCK and LCK and with MHC‐I, but negatively correlated with interferon and IgG in the CGGA (Figure 5A and 5) and TCGA datasets (Figure S8A and B). Additionally, CD204 was highly correlated with the immune checkpoint regulators PD‐L1 and TIM‐3 in gliomas.^19^, ^20^

### 3.6 CD204 is correlated with immune population in glioma microenvironment

**Figure 6:**
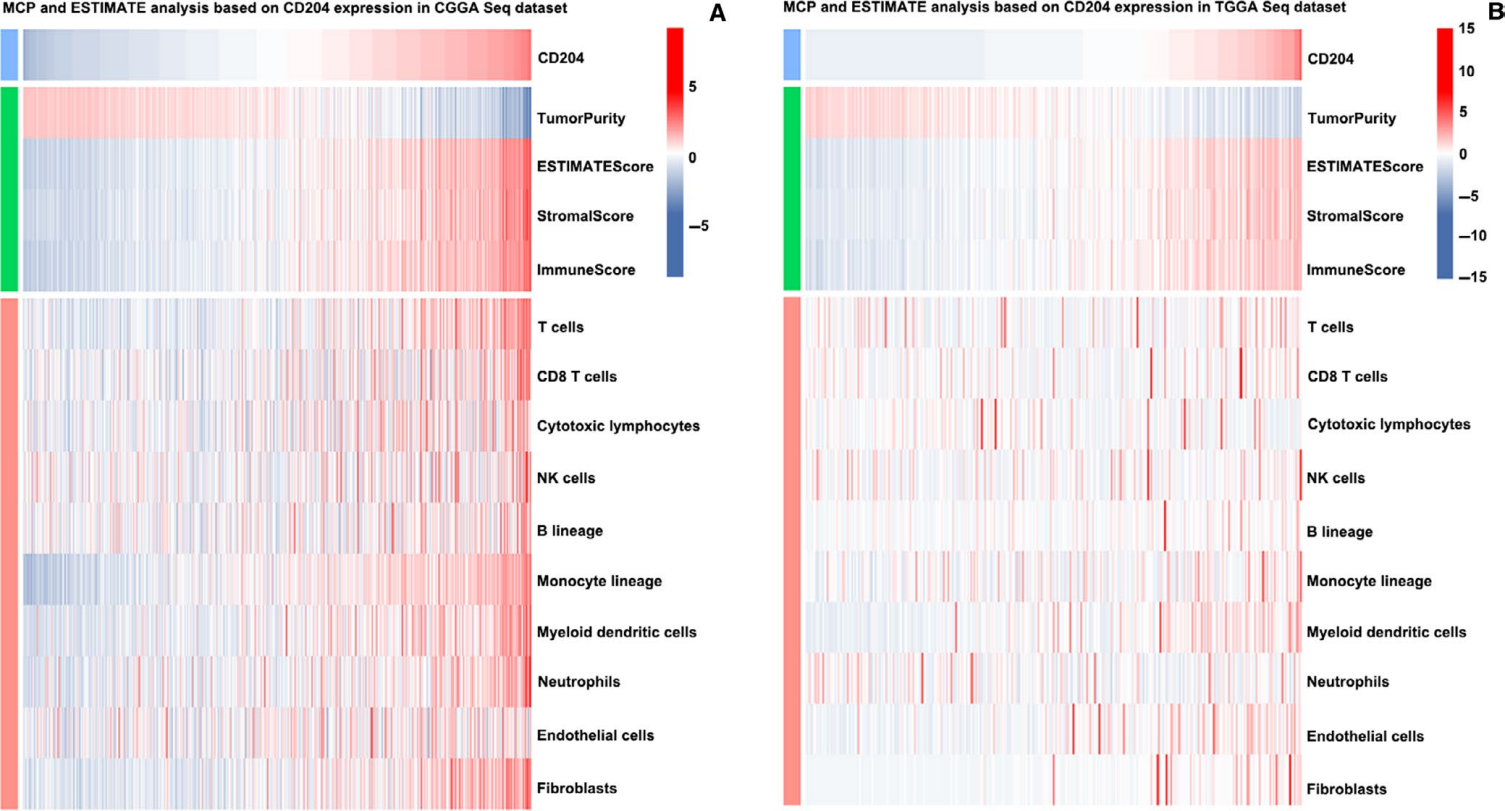
CD204 is closely associated with immune score and infiltrated cells in the glioma microenvironment. (A and B, upper panel) CD204 was positively associated with immune score and stromal score. The ESTIMATE method was used to evaluate the relationship between CD204 and immune populations. (A and B, lower panel) CD204 was closely associated with infiltrating cells in the tumor microenvironment. The MCP method was used to analyze the relationship between CD204 and immune populations

To fully understand the relationship between CD204 expression and immune cell infiltration, the MCP counter and ESTIMATE methods 21, 22 were used. The two methods consistently showed that CD204 was strongly associated with immune score (Figure 6A and 6, up panel) and immune cell population, especially with monocytic lineage, fibroblasts, and T cells (Figure 6A and 6, low panel).

### 3.7 CD204 is correlated with immune checkpoints

**Figure 7:**
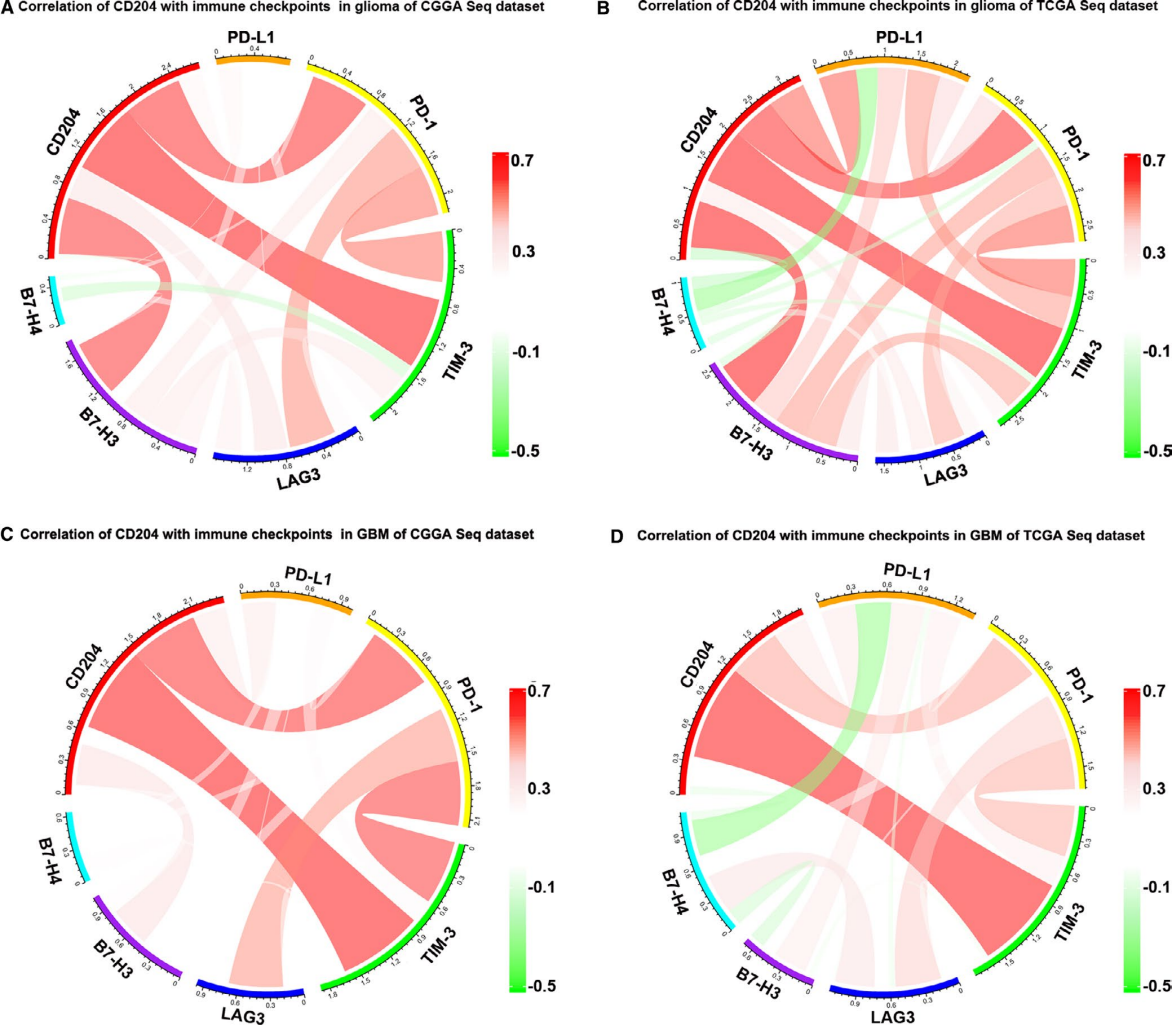
CD204 is correlated with immune checkpoint regulators. (A, B) Correlation of CD204 and immune checkpoints in whole‐grade glioma in CGGA and TCGA RNA‐seq data. CD204 and the mRNA expression levels of immune checkpoint‐related genes were selected in two datasets, and the correlation of these genes was analyzed by R and visualized using circos plots. (C, D) Correlation of CD204 and immune checkpoints in GBM in CGGA and TCGA RNA‐seq data

To assess whether CD204 targeting can be combined with immune checkpoint targeting, the expression of immune checkpoint‐related genes in glioma and GBM, namely CD274 (PD‐L1), PDCD1 (PD‐1), HAVCR2 (TIM‐3), LAG3, CD276 (B7‐H3), and VTCN1 (B7‐H4) was assessed in the CGGA and TCGA datasets. Pearson correlation analysis was performed to evaluate the association between CD204 and the immune checkpoint‐related genes. Interestingly, CD204 was significantly correlated with many immune checkpoints; of note, CD204 showed the strongest correlation with TIM‐3 and PD‐1 in both whole‐grade glioma (Figure 7A and 7) and GBM (Figure 7C and 7) and were further confirmed in our glioma samples (Figure S9). To make our work more reliable, we analyzed the expression and function of CD204^+^ cells in glioma tissues. We isolated CD14^+^CD204^+^ cells from glioma tissues, the basic expression of CD204 and sorting efficiency were shown in Figure S10. Then, the sorted cells were co‐cultured with CD8^+^ T cells from the same patient, and the results showed that CD204^+ ^cells significantly inhibit the function of T cells (Figure S11).

## 4 DISCUSSION

Major therapeutic strategies, including surgery, radiation, and chemotherapy, fail to cure glioma,^23^ thus warranting research of new therapeutic strategies. Recent advances in cancer immunotherapy have resulted in an increased efficiency in tumor treatment.^24^ In particular, immune checkpoint blockade has had great success in treatment of advanced cancers. In preclinical models, anti‐CTLA‐4 blockade alone or in combination with PD‐1 blockade significantly prolonged the long‐term survival of glioma patients.^25^ Likewise, anti‐PD‐1/anti‐PD‐L1 blocking antibody monotherapy also showed promising effects.^26^ Nevertheless, many patients still do not benefit from the treatments. It has been shown that many factors limit the efficiency of immunotherapy, one of which is the immunosuppressive tumor microenvironment.^27^

In the glioma microenvironment, TAMs constitute the majority of stromal cells. Although alternative strategies that target TAMs have had great success, many limitations still exist. For instance, reversing the polarization of TAMs through CSF‐1R inhibition increased the short‐term survival.^28^ However, this strategy does not yield an increased long‐term survival because TAMs induce resistance to CSF‐1R inhibition through secretion of IGF‐1 and subsequent activation of PI3K signaling in tumor cells.^29^ This has spurred interest in finding novel and specific targets, with the goal of realizing the full potential of immune checkpoint blockade.

Several molecules have been reported as specific markers of TAMs in glioma, including CD204, CD163, CD11b, CD14, and CD68. Interestingly, we found that CD204 was the only independent prognosticator for glioma. However, since its role in glioma is still scarcely understood, we aimed to elucidate it by analyzing microarray and RNA‐seq data in the CGGA and TCGA datasets. We observed that CD204 is correlated with other TAM markers. Next, survival analysis showed that high expression of CD204 predicted a significantly short OS in glioma. We also observed that CD204 expression was correlated with age and glioma grade and was significantly overexpressed in wild‐type IDH glioma, which has a shorter OS than mutant IDH glioma, indicating that TAMs are more prevalent in wild‐type IDH glioma and that CD204 may be used as a potential biomarker for these gliomas. Additionally, CD204 was also found to be highly expressed in the mesenchymal molecular subtype of glioma, and it showed high sensitivity and specificity, indicating that it may serve as a potential biomarker for mesenchymal glioblastoma. However, further clinical and basic studies are required to confirm these findings.

To get a better understanding of CD204, its biological function was explored using GO analysis: inflammatory response was the most enriched GO term. It is well known that, if tumor cells are not completely eliminated by effector T cells in the early phase, chronic inflammation will occur subsequently and negative regulatory mechanisms will limit T‐cell functions.^30^ This is consistent with our results that TAMs act as the most important contributors in initiating the inflammatory response to create a negative microenvironment, protecting tumor cells from immune destruction. Notably, the innate immune response, antigen processing and presentation, and MHC‐II activity, which exhibit antitumor functions, were also enriched. These findings impelled us to detect more precise markers to further identify the subtype of antitumor macrophages. CD204 was positively correlated with HCK and LCK and negatively correlated with IgG response. Apart from inflammatory response, immune response mediated by CD204 was also enriched. Thus, by evaluating the association of CD204 and infiltrating immune cells, we found that CD204 was correlated with the monocytic lineage, fibroblasts, and T cells, suggesting that TAMs suppress the activation of T cells by interacting with infiltrating cells in the tumor microenvironment.

Since the tumor microenvironment is the biggest obstacle to immunotherapy, and considering the importance of immune checkpoint blockade therapy in glioma, we evaluated the correlation between CD204 and immune checkpoint‐related genes. Indeed, CD204 showed high correlation with immune checkpoints, especially TIM‐3 and PD‐1, suggesting the possible synergy of CD204^+^ TAMs and immune checkpoint regulators in inhibiting the function of T cells in glioma. Reportedly, TIM‐3 triggers polarization of M2 macrophage by inhibiting STAT1 and promoting TGF‐β signaling pathway.^31^, ^32^ Moreover, blocking TIM‐3 rescued macrophage and T‐cell function, in vitro analysis also revealed that CD204^+^CD14^+^ cells significantly inhibited T‐cell function, suggesting that targeting CD204 and blocking TIM‐3 in glioma treatment might provide a considerable curative effect, especially in patients resistant to PD‐1/PD‐L1/CTLA‐4 therapy.^33^

In summary, to the best of our knowledge, this is the first study exploring the clinical expression and biological processes, including inflammatory and immune response of on a large sample of glioma. This study presents novel information key for understanding the role of CD204 and highlights it as a potential target for combined immunotherapy in glioma. These results will thus undoubtedly help to further optimize associated cancer immunotherapy.

## CONFLICT OF INTEREST

The authors declare no potential conflict of interest.
