# Supplementary material for: Characterization of transcriptome profile and clinical features of a novel immunotherapy target CD204 in diffuse glioma
Source: Cancer Med. 2019 May 29;8(8):3811–21. doi: 10.1002/cam4.2312 (PMC6639170; doi:10.1002/cam4.2312)
Supplement: Supplementary file 1 [file CAM4-8-3811-s001.pdf]

Supplementary Table 1. Primer sequences for qRT-PCR

Gene	Forward	Reverse
CD204	5’-GCAGTGGGATCACTTTCACAA-3’	5’-AGCTGTCATTGAGCGAGCATC-3’
PD-1	5’-GAGGGAATGCGTATTTTGGGT-3’	5’-AGGTTGTTCTTGTGTCACCTG-3’
TIM-3	5’-CTGCTGCTACTACTTACAAGGTC-3’	5’-GCAGGGCAGATAGGCATTCT-3’
GAPDH	5’-AGGAGCGAGATCCCTCCAAAAT-3’	5’-GGCTGTTGTCATACTTCTCATGG-3’

Supplementary Table 2. Univariate and multivariate Cox analysis of clinical prognostic parameters in TCGA. (n=411)

Characteristic	Univariate cox			Multivariate cox		
	**HR**	**95%CI**	***P***	**HR**	**95%CI**	***P***
Molecular subtypes	0.4872	0.4202-0.5648	<0.0001	0.9215	0.7525-1.1284	0.4289
IDH-mut	10.2344	6.9359-15.1016	<0.0001	4.1325	2.0301-8.4121	0.0001
Grade	11.2846	7.4781-17.0287	<0.0001	1.3786	0.7439-2.5549	0.3077
Age	6.6065	4.5501-9.5922	<0.0001	2.5194	1.6246-3.9071	<0.0001
Gender	0.9421	0.6678-1.3291	0.7343	0.7874	0.5390-1.1503	0.2165
Additional radiation therapy	0.6340	0.4481-0.8971	0.0101	0.8396	0.5434-1.2972	0.4309
MGMT promoter status	0.2853	0.1999-0.4072	<0.0001	1.0238	0.6720-1.5598	0.9127
CD204	1.6921	1.5251-1.8773	<0.0001	1.2714	1.1117-1.4540	0.0005

Abbreviations: HR, hazard ratio; CI, confidence interval; IDH-mut, isocitrate dehydrogenase mutation status; MGMT, methylguanine methyltransferase.

Supplementary Figure Legends

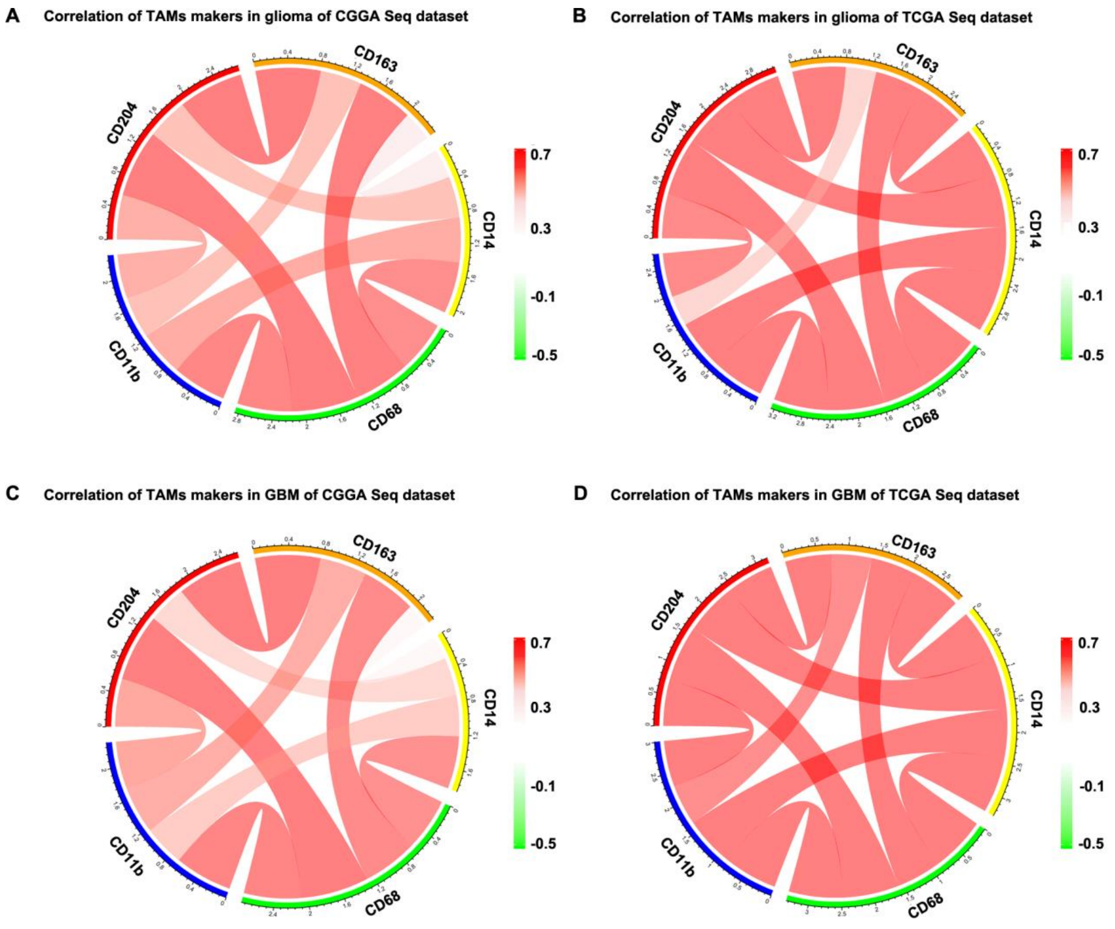

Figure S1. The correlation of TAM markers in glioma. (A, B) Correlation of TAM markers in glioma from CGGA and TCGA datasets. (C, D) Correlation of TAM markers in GBM from CGGA and TCGA datasets.

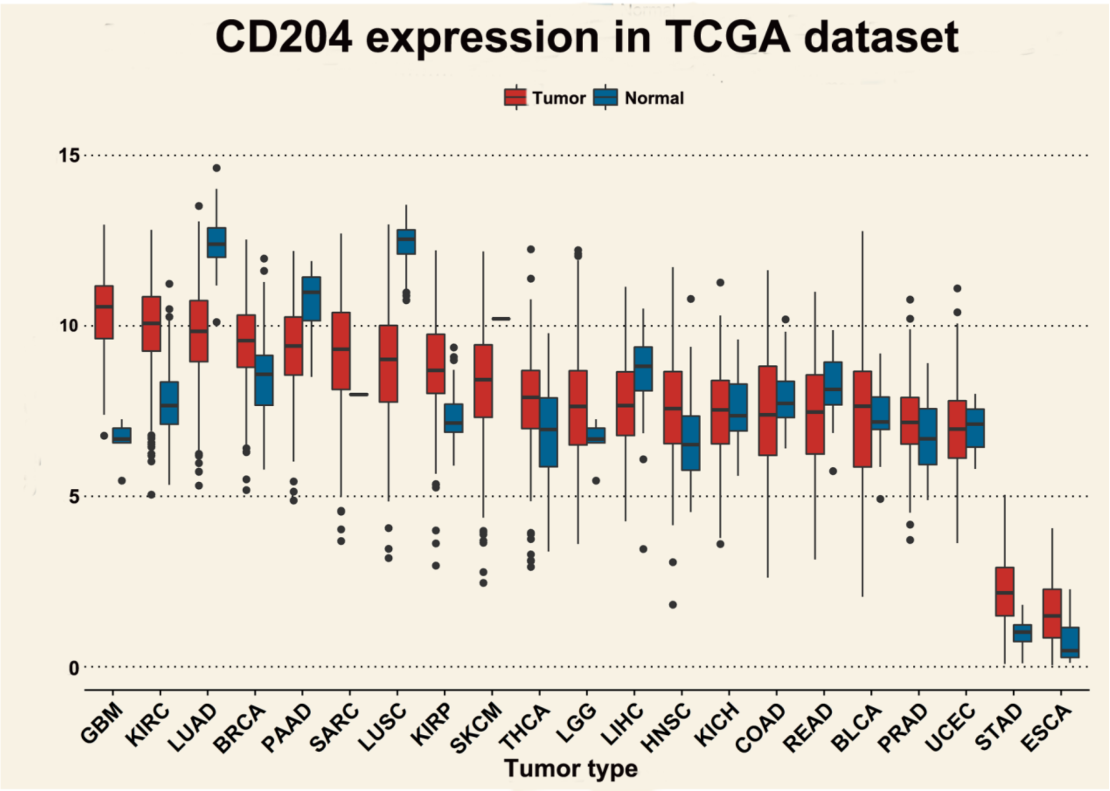

Figure S2. The landscape of CD204 expression in solid tumors from the TCGA dataset. CD204 had the highest expression in GBM.

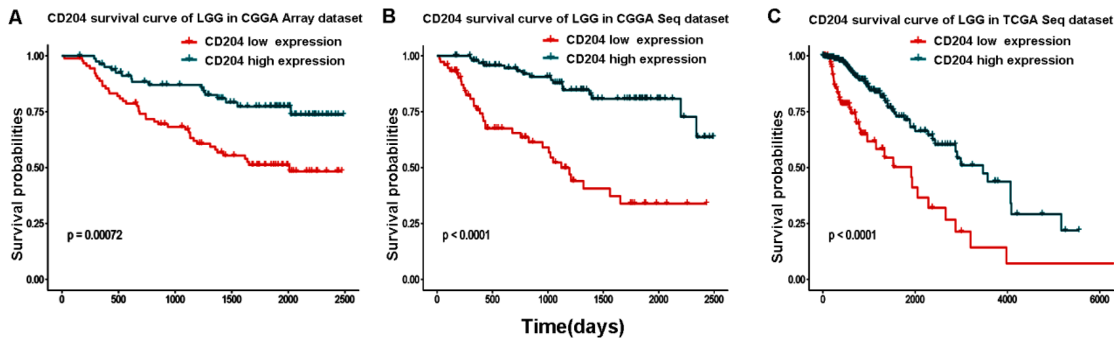

Figure S3. Figure 1. CD204 is correlated with unfavorable overall survival in low grade glioma. (A, B, C) High expression of CD204 was correlated with worse outcome in low grade glioma patients in the CGGA microarray and RNA-seq dataset and the TCGA RNA-seq dataset. The samples were divided into a high- and low-expression group based on the best cut off point of CD204 mRNA expression value.

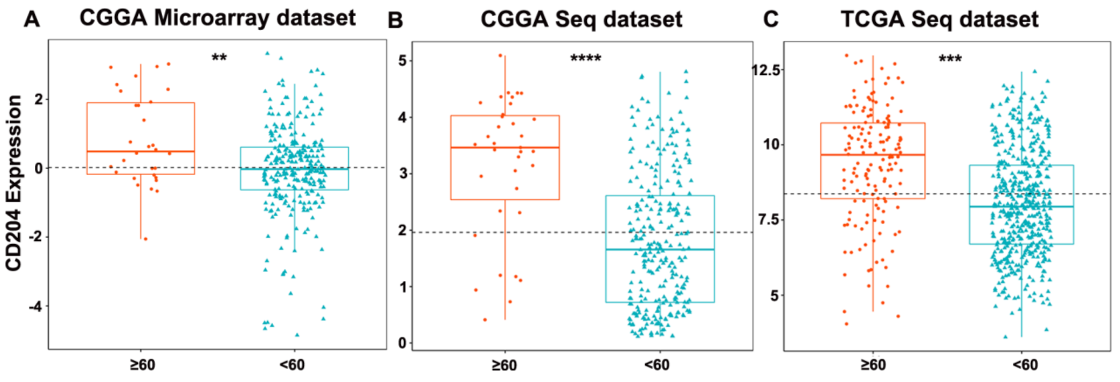

Figure S4. CD204 expression was correlated with age of glioma patients. Glioma patients were divided into two groups based on age, < 60 and ≥ 60, and the difference in CD204 expression between the groups was analyzed using R.

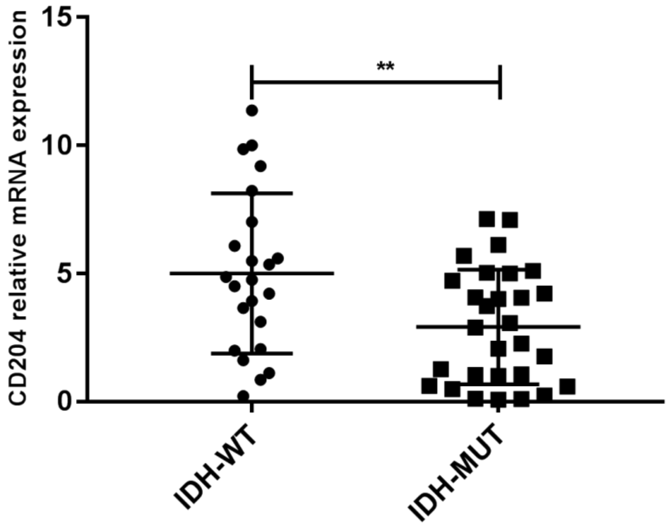

Figure S5. CD204 expression in IDH wild type and mutant glioma samples. The clinical glioma tissues were divided into two groups based on IDH status (IDH-WT=23, IDH-MUT=29), the CD204 expression level was detected by qRT-PCR. (* $P < 0.05$, ** $P < 0.01$, *** $P < 0.001$)

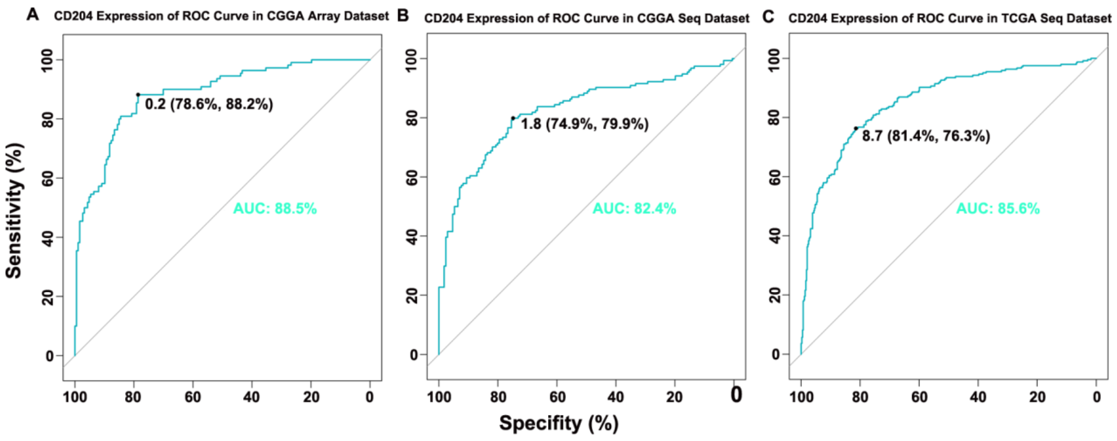

Figure S6. CD204 is a potential marker for wild-type IDH glioma. (A, B, C) ROC curve analysis of CD204 in wild-type IDH glioma by analyzing microarray and RNA-seq data in the CGGA dataset and RNA-seq data in the TCGA dataset.

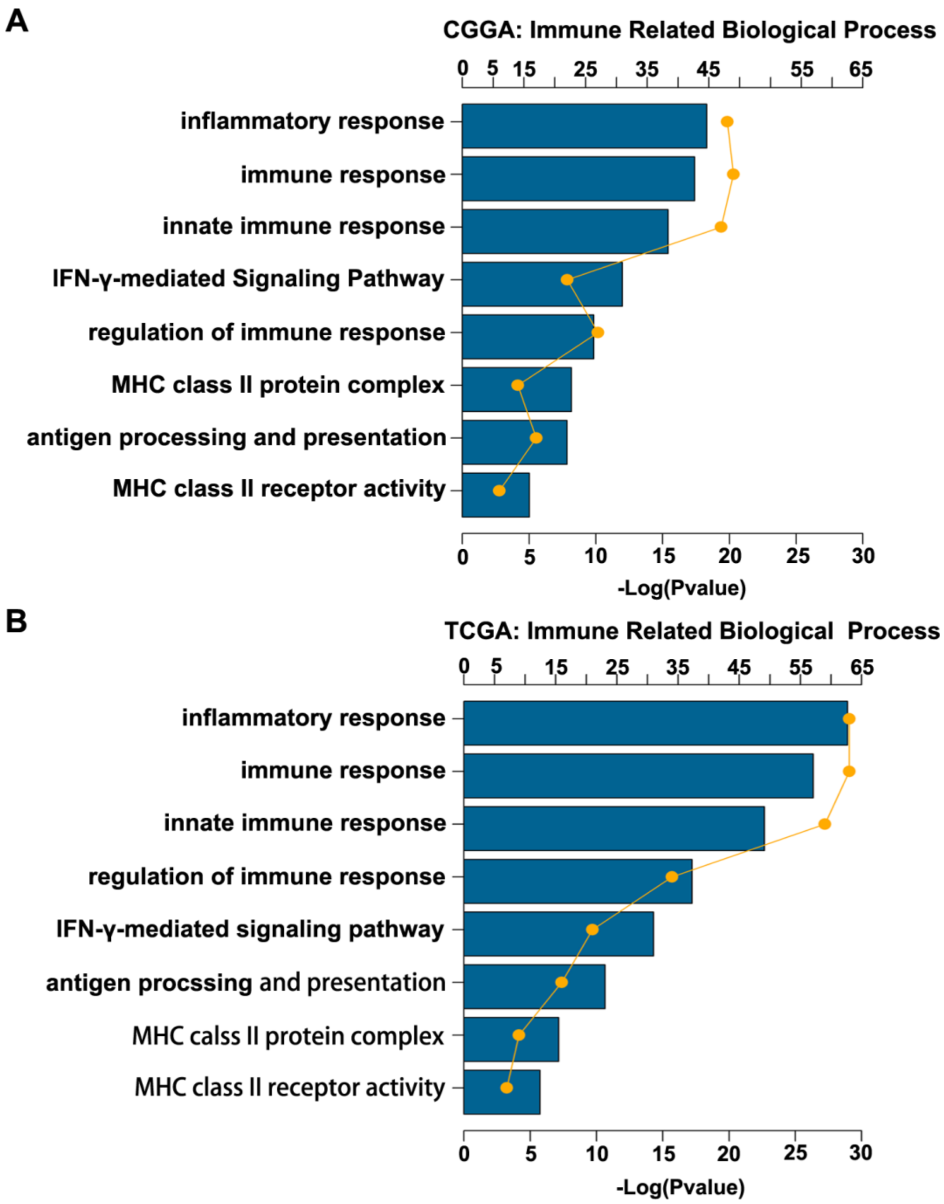

Figure S7. Biological functions of CD204. (A, B) Detailed information of CD204-related GO terms in the CGGA and TCGA datasets.

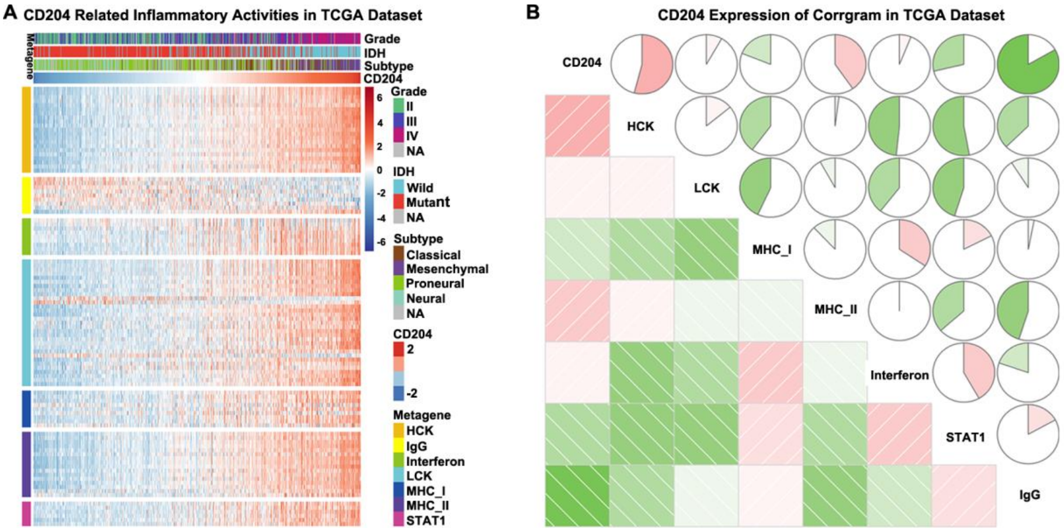

Figure S8. CD204 in inflammatory response in the TCGA dataset. (A) The heatmap of CD204-related inflammatory metagenes in the TCGA dataset. CD204 was positively correlated with HCK, LCK, and interferon and negatively correlated with IgG. (B) Correlogram of CD204 and inflammatory metagenes in the TCGA dataset.

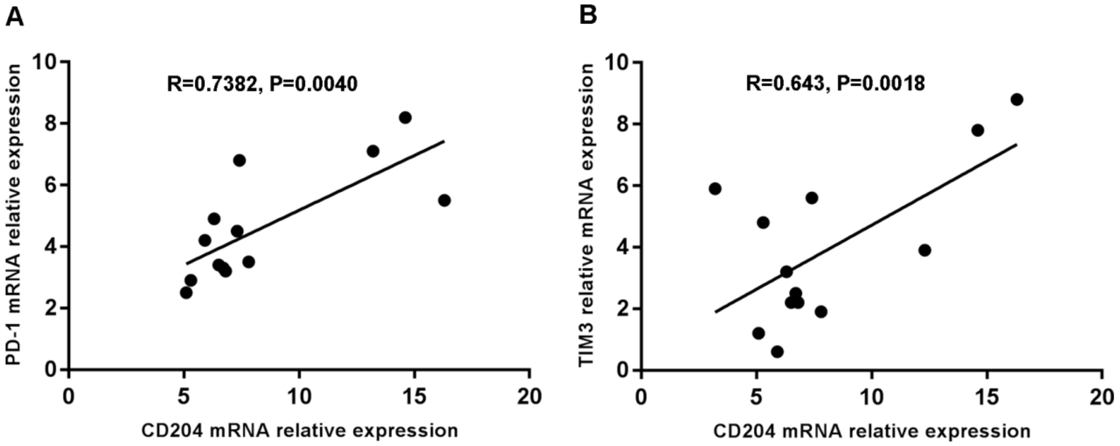

Figure S9. CD204 is correlated with PD-1 and TIM-3 in clinical samples. (A) CD204 positively correlated with PD-1. (B) CD204 positively correlated with TIM-3.

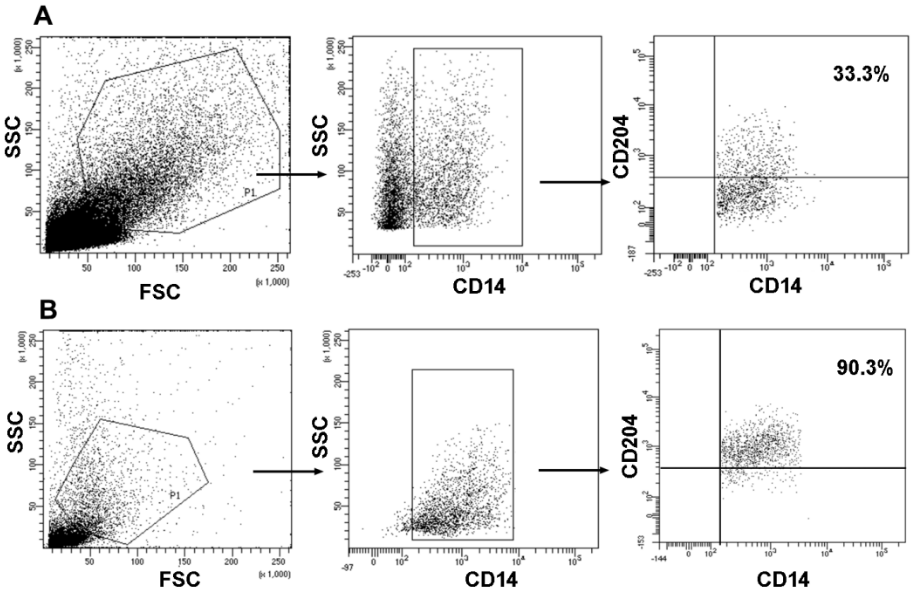

Figure S10. Sorting efficiency of CD14^{+}CD204^{+} cells. (A) Expression of CD204 on CD14^{+} in glioma samples. (B) The ratio of CD14^{+}CD204^{+} isolated from glioma tissue.

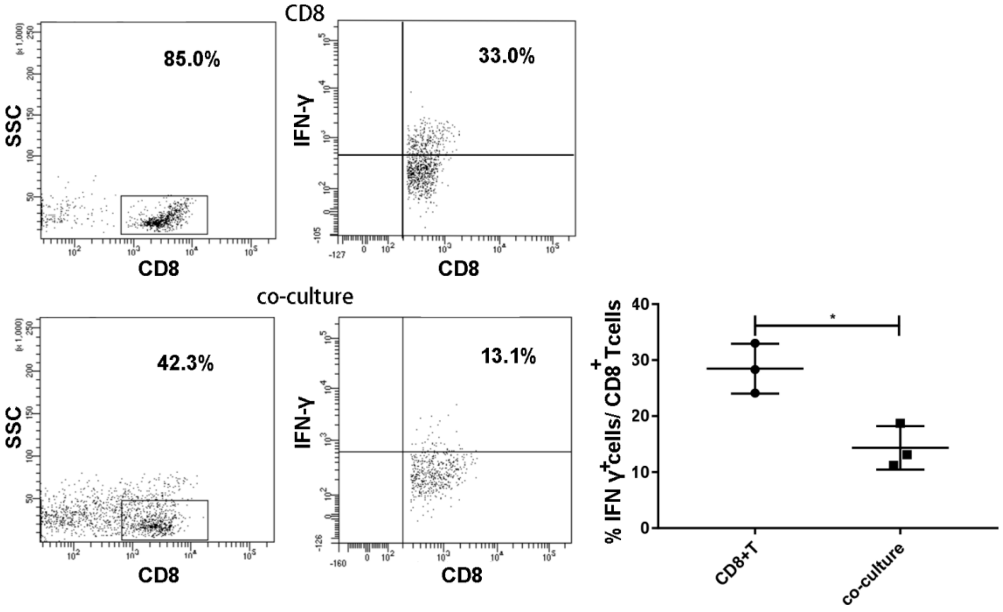

Figure S11. Analysis of T cell function. CD8+T cells were co-cultured with $CD14^{+}CD204^{+}$ cells, the IFNγ expression were analyzed by FACS. (* *P* ＜0.05, ** *P* ＜0.01, *** *P* ＜0.001)
